# Allele-specific polymerase chain reaction for the detection of Alzheimer’s disease-related single nucleotide polymorphisms

**DOI:** 10.1186/1471-2350-14-27

**Published:** 2013-02-19

**Authors:** Mohd Nazif Darawi, Chin Ai-Vyrn, Kalavathy Ramasamy, Philip Poi Jun Hua, Tan Maw Pin, Shahrul Bahyah Kamaruzzaman, Abu Bakar Abdul Majeed

**Affiliations:** 1 Brain Science Research Laboratory, Faculty of Pharmacy, Universiti Teknologi MARA, Puncak Alam, Selangor, 42300, Malaysia; 2 Ageing and Age Associated Disorders Research Group, Department of Medicine, Faculty of Medicine, University Malaya, Kuala Lumpur, 50603, Malaysia; 3 Collaborative Drug Discovery Research Group, Faculty of Pharmacy, Universiti Teknologi MARA, Puncak Alam, Selangor, 42300, Malaysia; 4 Research Management Institute, Universiti Teknologi MARA, Shah Alam, Selangor, 40450, Malaysia

**Keywords:** Alzheimer’s disease, Single nucleotide polymorphism, Apolipoprotein E, Bridging integrator, Clusterin, ATP-binding cassette sub-family A member 7, Complement receptor 1, Phosphatidylinositol binding clathrin assembly protein, Allele-specific polymerase chain reaction

## Abstract

**Background:**

The incidence of Alzheimer’s disease, particularly in developing countries, is expected to increase exponentially as the population ages. Continuing research in this area is essential in order to better understand this disease and develop strategies for treatment and prevention. Genome-wide association studies have identified several loci as genetic risk factors of AD aside from apolipoprotein E such as bridging integrator (*BIN1*), clusterin (*CLU*), ATP-binding cassette sub-family A member 7 (*ABCA7*), complement receptor 1 (*CR1*) and phosphatidylinositol binding clathrin assembly protein (*PICALM*). However genetic research in developing countries is often limited by lack of funding and expertise. This study therefore developed and validated a simple, cost effective polymerase chain reaction based technique to determine these single nucleotide polymorphisms.

**Methods:**

An allele-specific PCR method was developed to detect single nucleotide polymorphisms of *BIN1 rs744373*, *CLU rs11136000*, *ABCA7 rs3764650*, *CR1 rs3818361* and *PICALM rs3851179* in human DNA samples. Allele-specific primers were designed by using appropriate software to permit the PCR amplification only if the nucleotide at the 3’-end of the primer complemented the base at the wild-type or variant-type DNA sample. The primers were then searched for uniqueness using the Basic Local Alignment Search Tool search engine.

**Results:**

The assay was tested on a hundred samples and accurately detected the homozygous wild-type, homozygous variant-type and heterozygous of each SNP. Validation was by direct DNA sequencing.

**Conclusion:**

This method will enable researchers to carry out genetic polymorphism studies for genetic risk factors associated with late-onset Alzheimer’s disease (*BIN1, CLU, ABCA7, CR1* and *PICALM*) without the use of expensive instrumentation and reagents.

## Background

Alzheimer’s disease (AD) is the most common cause of dementia in the older population. The incidence and prevalence of dementia is projected to increase exponentially as the worldwide population ages. The estimated number of people living with dementia worldwide in 2009 was approximately 34.4 million. The total societal cost of dementia worldwide in that year was estimated to be US$422 billion [1]. More than 90% of all AD cases are late-onset AD (LOAD), the AD subtype in which symptoms appear after the age of 65 years [2]. Many studies have demonstrated associations between LOAD and genetic, lifestyle and environmental factors. Genetic factors, however, are likely to play a crucial role [3]. The strongest known genetic risk factor for LOAD is the ε4 allele of the *APOE* gene. A number of studies have identified *APOE ε4* as a genetic susceptibility factor for AD in different ethnic populations [4]. However, the impact of *APOE ε4* allele on LOAD is limited as the specificity and sensitivity of the genotype is only 68 and 65 percent respectively [5]. As such, other genetic risk markers are likely to play an important role in the development of LOAD.

The Alzgene database (http://www.alzgene.org) is a web-based overview of collective data, systematic meta-analyses, and regularly updated genetic association studies published in the field of AD research. A number of SNPs from different genes have been linked to AD through candidate gene association and genome-wide association (GWA) studies which have incorporated new high throughput and rapid scanning genotyping technologies not readily available in developing countries. The most common genes associated with LOAD in this database on the Human Genome Epidemiology Network (HuGENET) interim guidelines for the assessment of genetic association studies (updated 18th April 2011) include *APOE rs429358* and *rs7412, BIN1 rs744373, CLU rs11136000, ABCA7 rs3764650, CR1 rs3818361, PICALM rs3851179, MS4A6A rs610932, CD33 rs3865444, MS4A4E rs670139, and CD2AP rs9349407*[6].

Allele-specific polymerase chain reaction (AS-PCR), also known as amplification refractory mutation system (ARMS) or PCR amplification of specific alleles (PASA) is a PCR-based method which can be employed to detect the known SNPs [7]. The concept of AS-PCR was initiated by Newton et al. [8], approximately six years after PCR was invented. In this approach, the specific primers are designed to permit amplification by DNA polymerase only if the nucleotide at the 3’-end of the primer perfectly complements the base at the variant or wild-type sequences. After the PCR and electrophoresis, the patterns of specific PCR products permit the differentiation of the SNPs. Several innovative approaches have been employed to detect the presence of specific PCR product. Some are based on probe hybridization which requires specific labelled probes [9] and melting curve analysis [10] which requires nucleic acid stains. AS-PCR has been utilized widely in many areas of study such as pharmacogenetics [11], genetic disorders [12,13], microbiology [14] and others.

This concept in determining SNP is relatively cheaper than other available methods. Primer design and well-optimized PCR methodology are the crucial aspects in creating a working AS-PCR-based genotyping system. Once the optimized protocol has been achieved, the execution of AS-PCR is relatively simple, analogous to the conventional PCR. The AS-PCR for APOE genotyping was developed and utilized to differentiate the *ε2/ε3/ε4* genotype in 1991 [15]. An improved method was described in 1999 [16]. This study was therefore carried out to develop, validate and utilize an AS-PCR method to determine SNPs in the next five most common genes from the Alzgene database (*BIN1, CLU, ABCA7, CR1* and *PICALM*).

## Methods

### Ethics approval

The study was conducted with the approval of the Research Ethics Committee of Universiti Teknologi MARA (UiTM) and the Medical Ethics Committee of the University of Malaya Medical Centre (UMMC), which adheres to the Declaration of Helsinki.

### DNA samples

Subjects were recruited from the memory and general geriatric outpatient clinics of UMMC, Kuala Lumpur, Malaysia from July of 2011 to June of 2012. 5 mls of blood was collected from each subject after an informed consent was obtained from the subject or his/her guardian. Genomic DNA was extracted from the whole blood using a QIAamp DNA Blood Mini Kit (Qiagen, USA) according to the manufacturer’s instructions.

### Genotyping test

The genotyping method used to detect the selected SNPs variants was developed using AS-PCR. Primers were designed with the aid of Oligo Explorer 1.4 software and searched for uniqueness using the NCBI BLAST® search engine [17]. PCR was carried out using a thermal cycler (Eppendorf Mastercycler Gradient; Eppendorf, Hamburg, Germany). The final volume of all PCR protocols was 25 μL.

### First round PCR

The first round PCR comprised of 1X GoTaq® Green Master Mix (Promega Corp., WI, USA), 0.2 μM of forward common (Fc) and reverse common (Rc) primer of each gene, and approximately 50 ng of genomic DNA for the amplification of each gene. 1% of dimethyl sulphoxide (DMSO) was added only for the amplification of ABCA7. After the amplification, 2 μL of a 1:50 dilution of the first round PCR mixture was used in the second round PCR amplification (AS-PCR) using Fc, Rc, forward allele-specific (Fas) and reverse allele-specific primer (Ras). The concentration of primers in the second round PCR are shown in Table 1. The PCR cycling for the first and second round were the same.

**Table 1:** **Sequence of primers and the concentration for the genotyping of the polymorphism of *BIN1*, *CLU*, *ABCA7*, *CR1 *and *PICALM***

**Gene**	**SNP ID**	**Primer name**	**Sequence (5’-3’)**	**Concentration of primer in AS-PCR (μM)**
*BIN1*	*rs744373*	BIN1-Fc	AAG ACG GAG AGA GGA GGC AT	0.4
		BIN1-Rc	CCA TCT TCT TCT GCT CTC CCA G	0.1
		BIN1-Fas-W	CAT GGG CAG CCT CTG AG**A**	0.1
		BIN1-Ras-V	AGG CAG GTC TGA GGC **C**	0.1
*CLU*	*rs11136000*	CLU-Fc	CCT GGC TTA AAG AAT CCA CTC ATC	0.1
		CLU-Rc	CAG GGG ATT CCT TTG AGA TAG AGT	0.1
		CLU-Fas-W	GCA AGG GCC CGT TAG AGA **A**	0.1
		CLU-Ras-V	CAA AGC CAC ACC AGC TAT CAA AA**C**	0.1
*ABCA7*	*rs3764650*	ABCA7-Fc	AAA ATT AGC CAG GCG ACT TGG	0.05
		ABCA7-Rc	TCA GTG TCA CGG AGT AGA TCC	0.05
		ABCA7-Fas-W	GCT GCG AAC TTT GCA CC**T**	0.05
		ABCA7-Fas-V	GCT GCG AAC TTT GCA CC**G**	0.05
*CR1*	*rs3818361*	CR1-Fc	TGC TCC ATA ACC AGT AGT TGA A	0.1
		CR1-Rc	CAC TCA CCC TTC ATC GCA AA	0.1
		CR1-Ras-W	TGG GGC AAT TTC CTT TG**C**	0.4
		CR1-Fas-V	CCT CTG GTA AGC ATA AGA TAT A**A**	0.4
*PICALM*	*rs3851179*	PICALM-Fc	TCT ATT TTC TGC CTT ACT GTC	0.04
		PICALM-Rc	GCT GTT CAG TAA ATC TGA ATT TCT	0.04
		PICALM-Ras-W	CCA TAT AAT AGT TGT GAT AGA TAA **C**	0.3
		PICALM-Fas-V	CAA ACA ATA CAC ACT TCA GTA AAT **A**	0.04

The specific nucleotides at the 3’-end of primers were underlined.

### AS-PCR for *BIN1 rs744373*

The mixture of second round PCR comprised of 1X GoTaq® Green Master Mix, 2 μL of diluted first round PCR product, BIN1-Fc, BIN1-Rc, BIN1-Fas and BIN1-Ras primer. The PCR cycling was performed with an initial denaturation at 80°C for 5 minutes, followed by 35 cycles of amplification; 94°C for 1 min, 63°C for 30 s and 72°C for 46 s. The final extension was performed at 72°C for 5 min.

### AS-PCR for *CLU rs11136000*

The mixture of second round PCR comprised of 1X GoTaq® Green Master Mix, 2 μL of diluted first round PCR product, CLU-Fc, CLU-Rc, CLU-Fas and CLU-Ras primer. The PCR cycling for *CLU rs11136000* is the same as *BIN1 rs744373*.

### AS-PCR for *ABCA7 rs3764650*

The second round PCR required the use of two separate tubes for the amplification of wild-type and variant-type allele. The first tube containing the PCR mixture for the wild-type amplification comprised of 1X GoTaq® Green Master Mix, 2 μL of diluted first round PCR product, 1% of DMSO, ABCA7-Fc, ABCA7-Rc and ABCA7-Fas-W primer. The PCR mixture for the variant-type amplification comprised similar elements as in the first tube except for the ABCA7-Fas-W primer, which was replaced with the ABCA7-Fas-V primer. The PCR cycling was performed with an initial denaturation at 80°C for 5 minutes, followed by 35 cycles of amplification; 94°C for 1 min, 62°C for 30 s and 72°C for 51 s. The final extension was performed at 72°C for 5 min.

### AS-PCR for *CR1 rs3818361*

The second round PCR required the use of two separate tubes for the amplification of wild-type and variant-type allele. The first tube containing the PCR mixture for the wild-type amplification comprised of 1X GoTaq® Green Master Mix, 2 μL of diluted first round PCR product, CR1-Fc, CR1-Rc and CR1-Ras-W primer. The PCR mixture for the variant-type amplification comprised similar elements as in the first tube except for the CR1-Ras-W primer, which was replaced with the CR1-Fas-V primer. The PCR cycling was performed with an initial denaturation at 80°C for 5 minutes, followed by 35 cycles of amplification; 94°C for 1 min, 60°C for 30 s and 72°C for 40 s. The final extension was performed at 72°C for 5 min.

### AS-PCR for *PICALM rs3851179*

The second round PCR also required the use of two separate tubes for the amplification of wild-type and variant-type allele. The first tube containing the PCR mixture for the wild-type amplification comprised of 1X GoTaq® Green Master Mix, 2 μL of diluted first round PCR product, PICALM-Fc, PICALM-Rc and PICALM-Ras-W primer. The PCR mixture for the variant-type amplification comprised similar elements as in the first tube except for the PICALM-Ras-W primer, which was replaced with the PICALM-Fas-V primer. The PCR cycling was performed with an initial denaturation at 80°C for 5 minutes, followed by 35 cycles of amplification; 94°C for 1 min, 57°C for 30 s and 72°C for 43 s. The final extension was performed at 72°C for 5 min.

### Agarose gel electrophoresis

After the amplification, electrophoresis was performed at 100 V for 70 min in 1X tris-acetate-EDTA buffer on 1.5% agarose gel stained with ethidium bromide (0.5 μg/μL). The amplified PCR products were visualized under UV light.

### Validation and accuracy of method

The method was validated by direct DNA sequencing (First BASE Laboratory Sdn Bhd, Malaysia) using BigDye® Terminator v3.1 cycle sequencing kit chemistry (Applied Biosystems). The accuracy of this AS-PCR was verified by internal positive, external positive and external negative control in all PCR runs.

## Results

**Figure 1:**
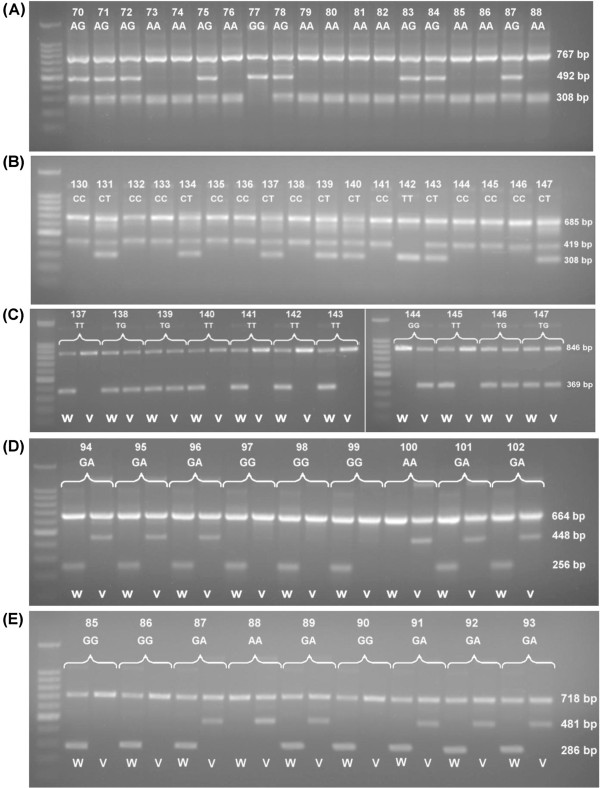
**The electrophoresis profiles for some of the successful amplifications.***BIN1 rs744373* (**A**), *CLU rs11136000* (**B**), *ABCA7 rs3764650* (**C**), *CR1 rs3818361* (**D**) and *PICALM rs3851179* (**E**). W = lane for wild-type amplification, V = lane for variant-type amplification.

**Figure 2:**
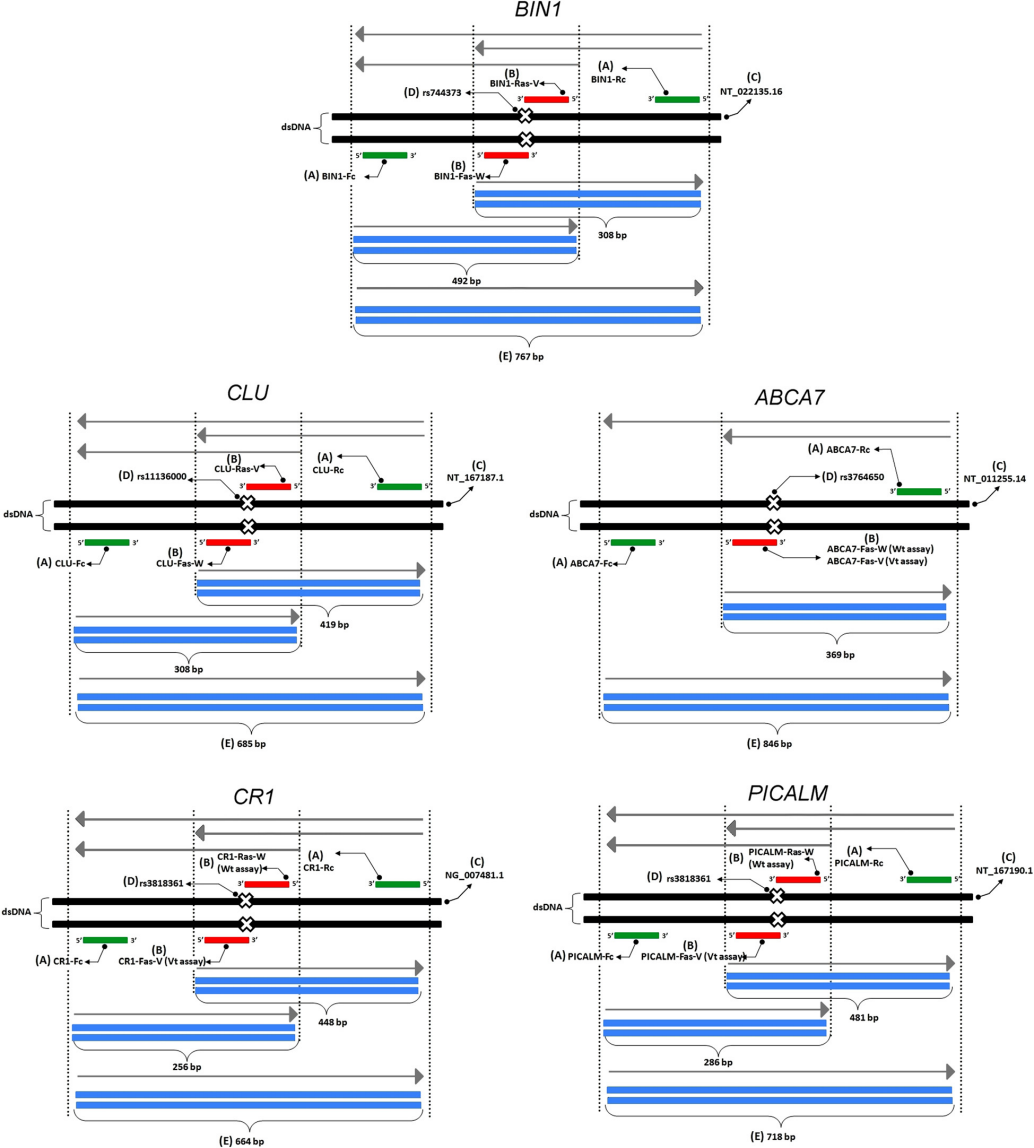
**Illustrations of PCR amplification and size of fragments.** The common primers (**A**) flank and amplify the non-specific-allele amplicons while the allele-specific primers (**B**) amplify the specific-allele amplicons to allow the differentiation of the genotypes. The DNA sequences were retrieved from GenBank (National Center for Biotechnology) using accession numbers (**C**). The non-specific-allele amplicons act as internal positive control (**E**). dsDNA = double-stranded DNA, Wt = wild-type, Vt = variant-type.

One hundred subjects were recruited for the study (mean age, 76.78 ± 6.1 years; range, 65–94 years, 61% female). Samples were genotyped for *BIN1 rs744373*, *CLU rs11136000*, *ABCA7 rs3764650*, *CR1 rs3818361* and *PICALM rs3851179*. The amplification and analysis of each SNP was performed successfully as shown in Figure 1. The presence of PCR bands with different sizes in the agarose gel indicated the genotype of the samples. Each reaction in different SNPs and genotypes are shown in detail in Table 2. The PCR amplifications, fragment size and accession number of DNA sequences are illustrated in Figure 2 [GenBank: NT_022135.16, NT_167187.1, NT_011255.14, NG_007481.1, and NT_167190.1]. The results that were obtained from the developed AS-PCR method were all consistent with genotype data obtained using a direct DNA sequencing technique.

**Table 2:** Different size of amplicons originated from different SNPs and genotypes

**Gene**	**Genotype**	**Assay**	**Interacted primers**		**Size of amplicons (bp)**
			**Forward**	**Reverse**	
*BIN1*	AA	Wild type and variant type	BIN1-Fc	BIN1-Rc	767
			BIN1-Fas-W		308
	AG		BIN1-Fc		767
			BIN1-Fas-W		308
			BIN1-Fc	BIN1-Ras-V	492
	GG			BIN1-Rc	767
				BIN1-Ras-V	492
*CLU*	CC	Wild type and variant type	CLU-Fc	CLU-Rc	685
			CLU-Fas-W		419
	CT		CLU-Fc		685
			CLU-Fas-W		419
			CLU-Fc	CLU-Ras-V	308
	TT		CLU-Fc	CLU-Rc	685
			CLU-Fc	CLU-Ras-V	308
*ABCA7*	TT	Wild type	ABCA7-Fc	ABCA7-Rc	846
			ABCA7-Fas-W		369
		Variant type	ABCA7-Fc		846
	TG	Wild type	ABCA7-Fc		846
			ABCA7-Fas-W		369
		Variant type	ABCA7-Fc		846
			ABCA7-Fas-V		369
	GG	Wild type	ABCA7-Fc		846
		Variant type	ABCA7-Fc		846
			ABCA7-Fas-V		369
*CR1*	GG	Wild type	CR1-Fc	CR1-Rc	664
				CR1-Ras-W	256
		Variant type		CR1-Rc	664
	GA	Wild type		CR1-Rc	664
				CR1-Ras-W	256
		Variant type		CR1-Rc	664
			CR1-Fas-V		448
	AA	Wild type	CR1-Fc		664
		Variant type	CR1-Fc		664
			CR1-Fas-V		448
*PICALM*	GG	Wild type	PICALM-Fc	PICALM-Rc	718
				PICALM-Ras-W	286
		Variant type		PICALM-Rc	718
	GA	Wild type		PICALM-Rc	718
				PICALM-Ras-W	286
		Variant type		PICALM-Rc	718
			PICALM-Fas-V		481
	AA	Wild type	PICALM-Fc		718
		Variant type	PICALM-Fc		718
			PICALM-Fas-V		481

## Discussion

Two common primers (Fc and Rc) were designed to flank and amplify the sequence containing the SNP. The PCR products obtained from this amplification are non-allele-specific amplicons as they are amplified constantly for any genotype sample. In the first round PCR, these amplicons can be used in the direct DNA sequencing method for AS-PCR validation purposes. In the second round PCR, these amplicons act as an internal positive control. Another pair of allele-specific primers (Fas and Ras) were designed to amplify allele-specific amplicons which were shorter fragments compared to the non-allele-specific amplicons (Figure 2). The nucleotide at the 3’-end of each allele-specific primer perfectly matched the SNP site. Fas-W or Ras-W and Fas-V or Ras-V primer were complementary to the wild-type and variant-type genotype sample respectively (Table 1).

The PCR was optimized by adjusting the concentration of each primer. The optimum annealing temperatures were determined using a gradient PCR. Under optimized PCR components and conditions, the patterns of PCR band that form after agarose gel electrophoresis allows the differentiation of the SNPs in order to determine whether the genotype was homozygous wild type, heterozygous or homozygous variant. The inclusion of an external negative control, a reaction containing all PCR components except the DNA sample was to confirm the absence of contamination and false positive results. The inclusion of an external positive control comprising the sequenced samples of each genotype was to observe the efficiency of the assay and false negative results. The genotyping test was further enhanced by the addition of a common PCR fragment acting as an internal positive control to guard against amplification failures and increase the specificity of the method.

The protocol for *ABCA7* genotyping required the use of DMSO as the DNA region of interest had a high GC content. Amplification of GC-rich regions of template is difficult due to the formation of secondary intramolecular structures as each GC pair is bound by three hydrogen bonds. DMSO has been reported to improve the amplification by interfering the self-complementarity of the DNA template and primers [18]. As such, in order to amplify this region which has 846 bp and 65.6% of GC content, a satisfactory yield of specific PCR products was obtained by including 1% of DMSO.

There are several methods that can be used to detect SNPs such as PCR restriction fragment length polymorphism (PCR-RFLP), high resolution melting (HRM), pyrosequencing and probe hybridization based techniques. PCR-RFLP has some disadvantages such as the necessity of an incubation period for enzymatic digestion by restriction endonuclease to separate the restriction fragments [19]. The other methods mentioned above are faster and easier to determine SNPs [20,21] but these methods are expensive because they require the use of high technology instrumentations and costly reagents.

The AS-PCR method developed in this study only requires basic equipment such as a conventional thermal cycler and a gel documentation system which are available in most genetic laboratories. It is cost–effective as it does not use fluorescent nucleic acid stains or hybridization probes, whilst retaining test sensitivity and specificity by the inclusion of positive and negative controls. This makes it suitable to be used in studies where lack of funding, equipment or expertise may be a factor.

## Conclusion

The use of this method will therefore enable researchers to carry out genetic polymorphism studies for genetic risk markers associated with LOAD (*BIN1, CLU, ABCA7, CR1 and PICALM*) without the use of expensive instrumentation and reagents.

## Competing interests

All authors declare that they have no financial and non-financial competing interests to report.

## Authors’ contributions

MND provided the conception and design of the study, supplied the acquisition of data, analysis and interpretation of data and drafting of manuscript. KR and CAV revised the article critically for important intellectual content and gave final approval of the version to be submitted. PPJH, TMP, SBK and ABAM were responsible for the article critically for important intellectual content. All authors have read and approve the final manuscript.

## Pre-publication history

The pre-publication history for this paper can be accessed here:

http://www.biomedcentral.com/1471-2350/14/27/prepub

## References

[B1] Wimo A Winblad B Jönsson L The worldwide societal costs of dementia: estimates for 2009 Alzheimers Dement 2010 6 98 103 10.1016/j.jalz.2010.01.010 20298969

[B2] Bekris LM Yu CE Bird TD Tsuang DW Genetics of Alzheimer’s disease J Geriat Psychiatry Neurol 2010 23 213 227 10.1177/0891988710383571 PMC3044597 21045163

[B3] Borenstein AR Copenhaver CI Mortimer JA Early-life risk factors for Alzheimer’s disease Alzheimer Dis Assoc Disord 2006 20 63 72 10.1097/01.wad.0000201854.62116.d7 16493239

[B4] Bertram L Tanzi RE Thirty years of Alzheimer’s disease genetics: the implications of systematic meta-analyses Nat Rev Neurosci 2008 9 768 778 10.1038/nrn2494 18802446

[B5] Mayeux R Saunders AM Shea S Mirra S Evans D Hyman BT Crain B Tang MX Phelps CH Utility of the apolipoprotein E genotype in the diagnosis of Alzheimer’s disease N Engl J Med 1998 338 506 511 10.1056/NEJM199802193380804 9468467

[B6] Bertram L McQueen MB Mullin K Blacker D Tanzi RE Systematic meta-analyses of Alzheimer’s disease genetic association studies: the AlzGene database Nat Genet 2007 39 17 23 10.1038/ng1934 17192785

[B7] Kwok PY Chen X Detection of single nucleotide polymorphisms Curr Issues Mol Biol 2003 5 43 60 12793528

[B8] Newton CR Graham A Hepstinstall LE Powell SJ Summers C Kalsheker N Smith JC Markham AF Analysis of any point mutation in DNA. The amplification refractory mutation system (ARMS) Nucleic Acids Res 1989 17 2503 2516 10.1093/nar/17.7.2503 2785681 PMC317639

[B9] Myakishev MV Khiripin Y Hu S Hamer HD High-throughput SNP genotyping by allele-specific PCR with universal energy-transfer-labeled primers Genome Res 2001 11 163 169 10.1101/gr.157901 11156625 PMC311033

[B10] Germer S Higuchi R Single-tube genotyping without oligonucleotide probes Genome Res 1999 9 72 78 9927486 PMC310703

[B11] Teh LK Lee WL Amir J Salleh MZ Ismail R Single step PCR for detection of allelic variation of MDR1 gene (P-glycoprotein) among three ethnic groups in Malaysia J J Clin Pharm Ther 2007 32 313 319 10.1111/j.1365-2710.2007.00822.x 17489883

[B12] Chen Q Lu P Jones AV Cross NC Silver RT Wang L Amplification refractory mutation system, a highly sensitive and simple polymerase chain reaction assay, for the detection of JAK2 V617F mutation in chronic myeloproliferative disorders J Mol Diagn 2007 9 272 276 10.2353/jmoldx.2007.060133 17384221 PMC1867436

[B13] Mirasena S Shimbhu D Sanguansermsri M Sanguansermsri T Detection of beta-thalassemia mutations using a multiplex amplification refractory mutation system assay Hemoglobin 2008 32 403 409 10.1080/03630260701798391 18654891

[B14] Sapkota BR Ranjit C Neupane KD Macdonald M Development and evaluation of a novel multipleprimer PCR amplification refractory mutation system for the rapid detection of mutations conferring rifampicin resistance in codon 425 of the rpoB gene of mycobacterium leprae J Med Microbiol 2008 57 179 184 10.1099/jmm.0.47534-0 18201983

[B15] Wenham PR Newton CR Price WH Analysis of apolipoprotein E genotypes by the amplification refractory mutation system Clin Chem 1991 37 241 244 1993332

[B16] Donohoe GG Salomaki A Lehtimaki T Pulkki K Kairisto V Rapid identification of apolipoprotein E genotypes by multiplex amplification refractory mutation system PCR and capillary gel electrophoresis Clin Chem 1999 45 143 146 9895356

[B17] Altschul SF Gish W Miller W Myers EW Lipman DJ Basic local alignment search tool J Mol Biol 1990 215 403 410 2231712 10.1016/S0022-2836(05)80360-2

[B18] Mammedov TG Pienaar E Whitney SE TerMaat JR Carvill G Goliath R Subramaniam A Viljoen HJ A fundamental study of the PCR amplification of GC-rich DNA templates Comput Biol Chem 2008 32 452 457 10.1016/j.compbiolchem.2008.07.021 18760969 PMC2727727

[B19] Hixson JE Vernier DT Restriction isotyping of human apolipoprotein E by gene amplification and cleavage with Hhal J Lipid Res 1990 31 545 548 2341813

[B20] Koch W Ehrenhaft A Griesser K Pfeufer A Müller J Schömig A Kastrati A TaqMan systems for genotyping of disease-related polymorphisms present in the gene encoding apolipoprotein E Clin Chem Lab Med 2002 40 1123 1131 12521230 10.1515/CCLM.2002.197

[B21] Aydin A Toliat MR Bähring S Becker C Nürnberg P New universal primers facilitate pyrosequencing Electrophoresis 2006 27 394 397 10.1002/elps.200500467 16331584

